# Analysis of the *VSX1* gene in keratoconus patients from Saudi Arabia

**Published:** 2011-03-08

**Authors:** Khaled K. Abu-Amero, Hatem Kalantan, Abdulrahman M. Al-Muammar

**Affiliations:** 1 Ophthalmic Genetics Laboratory, Department of Ophthalmology, College of Medicine, King Saud University, Riyadh, Saudi Arabia; 2 Anterior Segment Unit, Department of Ophthalmology, College of Medicine, King Saud University, Riyadh, Saudi Arabia

## Abstract

**Purpose:**

To screen the visual system homebox 1 (*VSX1*) gene in Saudi Arabian keratoconus patients.

**Methods:**

We sequenced the entire coding region, exon-intron boundaries in clinically confirmed keratoconus patients (n=55) and 50 ethnically matched healthy controls. All cases and controls were unrelated.

**Results:**

Sequencing *VSX1* revealed the presence of five nucleotide changes, 3 of which were non-coding (g.8326 G>A, g.10945 G>T, and g.11059 A>C) and 2 were synonymous-coding sequence changes (g.5053 G>T and g.8222 A>G). All five sequence changes were benign polymorphisms with no apparent clinical significance.

**Conclusions:**

In our keratoconus cohort, no pathogenic *VSX1* mutation(s) were identified.

## Introduction

Keratoconus (KTCN; OMIM 148300) is a non-inflammatory thinning and anterior protrusion of the cornea that results in steepening and distortion of the cornea, altered refractive powers and altered visual acuity. In more advanced cases, corneal scarring from corneal edema and decompensation further reduces visual acuity. Symptoms are highly variable and depend on the stage of progression of the disorder [1,2]. The prevalence of keratoconus has been reported to vary in different studies, from 8.8 to 54.4 per 100,000 [3,4], the variation is in part due to the different diagnostic criteria used. The incidence of keratoconus ranges between 1/500 to 1/2,000 individuals throughout the world [5]. The disease occurs with no ethnic or gender preponderance and causes significant visual impairment [2,5,6]. Keratoconus should be divided into three broad categories: i) keratoconus associated with rare genetic disorders (such as Down syndrome, nail-patella syndrome, neurofibromatosis, etc); ii) keratoconus in the setting of commonly reported associations (contact lens wear, eye rubbing, atopy, Leber congenital amaurosis, mitral valve prolapsed and positive family history) and iii) isolated keratoconus with no associations. Most cases of keratoconus are sporadic but some (5%–10%) have a positive family history [6,7]. In such cases both autosomal recessive and dominant patterns of inheritance have been reported [8-11]. There are several chromosomal loci and genes reported to be associated with keratoconus [6,11]. some of which were eventually excluded [6,12], while others showed no confirmed association with the disease [13,14]. This is not the case for the visual system homebox 1 (*VSX1*) gene where mutations associated with keratoconus cases have been found in different studies [15-18], although other studies which did not find *VSX1* mutations in cohorts of keratoconus patients from various populations [19,20]. This indicates that keratoconus is a complex condition of multi-factorial etiology and that mutations in *VSX1* are not responsible for all cases of keratoconus. To our knowledge, this is the first time *VSX1* was screened for mutations in the Saudi keratconus patients.

## Methods

### Patients and controls

The study adheres to the tenets of the Declaration of Helsinki, and all participants signed an informed consent. The study was approved by the College of Medicine (King Saud University, Riyadh, Saudi Arabia) ethical committee (proposal number # 09–659). All study subjects were self identified of Saudi Arabian ethnicity. Family names were all present in the database of Arab families of Saudi Arabian origin. In this study we screened 55 unrelated Saudi Keratoconus patients (each patient represents one family) and 50 ethnically matched unrelated controls for mutations in the *VSX1* gene. Patients were selected from the anterior segment clinic at King Abdulaziz University Hospital after examination. Patients were diagnosed with keratoconus if the Schimpff-flow based elevation map showed posterior corneal elevation within the central 5 mm ≥+20 µm, inferior-superior dioptric asymmetry (I-S value) >1.2 diopters (D) and the steepest keratometrey >47 D. Patients were considered as sporadic cases after examining the immediate family members and identifying the patient as isolated case of keratoconus. Exclusion criteria was based on presence of post- laser-assisted in situ keratomileusis (LASIK) ectasia and refusal to participate.

Controls were recruited from the general ophthalmology clinic and had no ocular disease(s) or previous ophthalmic surgeries. Their slit lamp exam showed clear cornea and their Schimpff-flow based elevation map were within normal limits.

All Keratoconus cases secondary to causes such as trauma, surgery, Ehlers Danlos syndrome, Osteogenesis Imperfecta and pellucid marginal degeneration were excluded from the study.

### DNA analysis

Five milliliters of peripheral blood were collected in EDTA tubes from all participating individuals. DNA was extracted using the illustra blood genomicPrep Mini Spin Kit from GE Healthcare (Buckinhamshire, UK), and stored at –20 °C in aliquots until required. PCR amplification of the *VSX1* coding region were performed using the primers detailed in Table 1. Successfully amplified fragments were sequenced in both directions using the M13 forward and reverse primers and the BigDye terminator v3.1 cycle sequencing kit (Applied Biosystems, Foster city, CA). Fragments were then run on the 3130xl Genetic Analyzer (Applied Biosystems) according to the manufacturer protocol. All sequenced fragments were analyzed using SeqScape software v2.6 (Applied Biosystems) and compared to the *VSX1* reference sequence (GenBank NG_008101).

**Table 1:** Primer sequences, PCR annealing temperature, and amplicon size for *VSX1* PCR amplification.

**Exon**	**Primer sequence**	**Annealing temperature (°C)**
VSX1–1F	**TGTAAAACGACGGCCAGT**CAGCTGATTGGAGCCCTTC	60
VSX1–1R	**CAGGAAACAGCTATGACC**GGGGCGATGGTCTGTGAC	
VSX1–2F	**TGTAAAACGACGGCCAGT**CCCAAGAGGTTCATAACTTCAA	60
VSX1–2R	**CAGGAAACAGCTATGACC**TATCATGCCGGGCCATAAAT	
VSX1–3aF	**TGTAAAACGACGGCCAGT**CTGGACAGCAGAGGAAGCA	60
VSX1–3aR	**CAGGAAACAGCTATGACC**TTGGCTATAGAGAAGGGACTGC	
VSX1–3bF	**TGTAAAACGACGGCCAGT**TGGACAGCAGAGGAAGCAG	60
VSX1–3bR	**CAGGAAACAGCTATGACC**TCAGGGGCAGATAATATACTCCA	
VSX1–4F	**TGTAAAACGACGGCCAGT**GCCCCTCTTCACTGTCTCTTC	60
VSX1–4R	**CAGGAAACAGCTATGACC**TTGCTTTGCTTTGGAAATGG	
VSX1–5aF	**TGTAAAACGACGGCCAGT**TGGGATTTAGAGAACAATAAGAAGG	58
VSX1–5aR	**CAGGAAACAGCTATGACC**CAGTGAAATCATTTTTGAACTTCG	
VSX1–5bF	**TGTAAAACGACGGCCAGT**CAGATGAGTGACCTAGGGAAAA	57
VSX1–5bR	**CAGGAAACAGCTATGACC**TGTCACATGTCCCCAGATTG	

F: Forward; R: Reverse; Bold sequences are those of the M13.

## Results

Fifty-five unrelated Keratoconus patients (Table 2) and 50 unrelated controls were recruited into this study. Of the 55 Keratoconus patients there were 24 males and 31 females with a mean age of 28.9 (SD 7.7). Of the 50 controls there were 19 males and 31 females with a mean age of 60 (SD 17). Examining the family pedigrees in the keratoconus patients indicated that the mode of inheritance was sporadic in 56.4% of cases, 34.6% were autosomal recessive; 5.4% were autosomal dominant, and 3.6% of cases were indeterminable **(**Table 2**)**. The full coding region, exon-intron boundaries and the 5′UTR and 3′UTR of *VSX1* was sequenced in all subjects. We detected five nucleotide changes in both patients and controls (Table 3). Two were previously reported (rs8123716 and rs12480307; and three are novel; Table 3). Three of the sequence changes g.5053 G>T, g.8326 G>A, and g.11059 A>C were heterozygous and two were homozygous (g.8222 A>G and g.10945 G>T). None of the sequence changes detected were pathogenic. Two (g.5053 G>T and g.8222 A>G) were synonymous coding, one intronic (g.8326 G>A), and two (g.10945 G>T and g.11059 A>C) in the 3′ UTR region.

**Table 2:** Clinical phenotypes of keratoconus patients.

**Patient demographics**			**Uncorrected visual acuity in Snellen's chart**		**Munsen sign**		**Vogt's striae**		**Hydrops**		**Scarring**		**Average Keratometry in VKG (in Diopters)**		**Optical pachymetry (mm)**		
**I.D.**	**Age**	**Sex**	**OD**	**OS**	**OD**	**OS**	**OD**	**OS**	**OD**	**OS**	**OD**	**OS**	**OD**	**OS**	**OD**	**OS**	**Mode of Inheritance**
1	20	M	20/100	20/200	+	+	+	+	+	+	-	+	51.9	64.7	525	502	SP
2	37	F	20/200	20/400	+	+	+	+	-	-	+	+	68.6	81.3	282	160	AR
3	18	M	CF	20/80	-	-	+	+	+	+	+	-	58	56	300	346	SP
4	17	M	CF	20/100	+	-	+	+	-	-	-	-	60	56.6	455	509	SP
5	35	F	20/20	20/60	-	-	-	+	-	+	-	+	43.1	44.6	584	554	SP
6	30	M	20/200	CF	-	-	-	-	-	-	-	-	49.1	68.7	434	349	SP
7	36	M	20/100	20/100	-	-	-	-	+	+	-	-	48.4	50.4	459	439	SP
8	16	M	CF	CF	+	+	+	+	+	+	-	+	54.4	55	241	268	SP
9	24	M	CF	20/80	+	+	+	+	+	+	+	+	53	65.7	302	397	SP
10	25	F	CF	CF	+	+	+	+	-	-	+	-	67.3	70.7	236	216	SP
11	25	M	20/20	CF	-	+	-	+	-	+	-	-	43.1	50.9	419	407	SP
12	46	F	20/80	20/100	-	-	+	+	+	+	-	-	53	57.6	463	412	ND
13	32	F	20/40	20/40	+	+	+	+	+	+	-	-	43.5	51.7	442	398	SP
14	24	F	20/100	20/60	-	-	+	+	-	+	-	-	43.2	45.8	484	464	AR
15	30	F	20/20	20/100	-	-	+	+	-	+	-	-	44.6	46.9	493	397	AR
16	20	F	20/25	20/100	-	-	-	+	-	+	-	-	42.7	56.2	509	456	SP
17	32	F	CF	20/200	-	-	-	-	+	+	-	-	56.3	52.6	429	471	SP
18	17	F	CF	20/25	+	+	+	-	+	+	+	+	69.3	45.5	284	522	SP
19	24	F	20/200	CF	-	+	-	+	+	+	-	+	49.8	62.2	492	218	SP
20	39	F	20/200	20/200	-	-	-	-	+	+	-	-	49.2	53.1	458	419	SP
21	28	F	CF	CF	+	+	+	-	+	+	+	+	67.6	61.8	327	287	AR
22	40	F	20/60	20/100	-	-	-	-	-	+	-	-	45.8	46.2	538	520	AD
23	32	F	20/60	CF	-	+	+	+	+	+	-	+	48.7	43.5	427	558	AD
24	25	M	20/40	CF	+	+	+	+	+	+	-	+	42.8	43.7	482	511	SP
25	17	F	20/60	20/100	+	+	-	+	+	+	-	-	56.4	58.2	411	414	SP
26	32	F	20/100	20/100	+	+	-	-	+	+	-	-	47.3	50.4	459	435	AR
27	22	F	20/100	20/100	-	-	-	-	+	+	-	-	46.4	44.5	425	416	SP
28	40	M	20/100	CF	+	+	+	+	+	+	+	-	69.2	71.8	262	359	AR
29	23	F	20/20	20/20	-	-	-	-	-	-	-	-	41.8	41.8	527	526	ND
30	35	F	CF	CF	-	-	-	-	-	-	-	-	43.6	63.1	482	371	SP
31	24	M	CF	CF	+	+	-	-	+	+	-	-	47.5	66	589	158	AR
32	25	M	CF	CF	+	+	+	+	+	+	+	+	61.8	62.6	384	379	AR
33	35	F	20/200	20/200	-	-	-	-	-	-	-	-	55.5	54.1	434	448	AR
34	25	M	20/30	20/100	-	+	-	+	-	+	-	-	44.2	63.2	477	405	AR
35	22	M	20/80	20/30	-	-	-	-	+	+	-	-	46.6	43.9	514	552	SP
36	25	M	20/200	CF	-	+	-	+	-	+	-	+	46	49	582	397	AR
37	30	M	20/30	CF	-	-	-	-	-	-	-	-	44	54.6	300	300	SP
38	40	F	20/200	CF	-	-	-	-	-	-	-	-	57.1	56.6	441	457	AR
39	28	M	20/200	20/100	-	-	-	-	-	-	-	-	50	57.7	435	388	AR
40	30	M	20/200	20/200	-	-	-	-	-	-	-	-	46.6	46.2	519	509	AR
41	29	F	20/100	20/40	-	-	-	-	+	+	-	-	47.4	43.6	446	495	SP
42	32	F	20/28	20/20	-	-	-	-	-	-	-	-	46	46.8	491	471	SP
43	29	F	20/200	20/100	-	-	-	-	-	-	-	-	47.8	46.4	516	512	SP
44	29	F	20/28	20/60	-	-	-	-	-	-	-	-	58.7	53.7	246	374	AD
45	24	M	20/200	20/60	-	-	-	-	+	+	-	-	49.8	45.9	467	487	SP
46	35	F	20/28	CF	-	-	-	-	-	+	-	-	47	47.1	546	508	SP
47	24	F	20/20	20/28	-	-	-	-	-	+	-	-	42.4	44.1	511	470	SP
48	40	F	CF	20/30	+	+	+	+	+	+	+	+	53.5	52	422	421	SP
49	23	M	20/200	20/80	-	-	-	-	-	-	-	-	57.9	47.3	365	464	AR
50	22	M	20/100	CF	-	+	-	+	+	+	-	+	45.6	68.5	491	381	SP
51	28	M	20/80	20/80	+	+	+	+	+	+	-	-	45.9	42.6	441	453	AR
52	41	M	20/28	20/100	-	-	-	-	-	-	-	-	43.3	46.4	494	474	SP
53	50	M	20/40	20/40	+	+	+	+	+	+	-	-	42.9	43.4	492	476	AR
54	40	F	CF	CF	+	+	+	+	+	+	+	+	57.6	53.4	225	242	AR
55	23	F	20/60	20/20	-	-	-	-	+	-	-	-	48.1	44.7	465	515	AR

Key: M=Male; F=Female; OD=Right eye; OS=Left eye; +=Positive; -=Negative; VKG=Videokeratography; AD=Autosomal dominant; AR=Autosomal recessive; SP=Sporadic; ND=not determined due to difficulty in predicting the mode of inheritance from available family pedigree. *Mode of inheritance was established (when possible) by examining the family pedigree carefully and taking detailed family history up to 2–3 generations.

**Table 3:** *VSX1* sequence changes observed in keratoconus patients and controls.

**Nucleotide change**	**Codon change**	**Location on the gene**	**Patients (n=55)**	**Controls (n=50)**
g.5053 G>T	p.S6S	Exon 1	2 (3.6%)	2 (4%)
g.8222 A>G	p.A182A	Exon 3	1 (1.8%)	1 (2%)
g.8326 G>A	Non-coding	Intron 3	1 (1.8%)	5 (10%)
g.10945 G>T	Non-coding	3′UTR	0	1 (2%)
g.11059 A>C	Non-coding	3′UTR	1 (1.8%)	0

Key: UTR=untranslated region. Nucleotide are numbered as in GenBank accession number NG_008101.

## Discussion

We detected five nucleotide changes in both the patients and control groups-none of which were pathogenic. Similar results have been published recently [19]. The lack of pathogenic sequence changes in *VSX1* in our keratoconus cohort, indicates that *VSX1* mutations are not responsible for keratoconus cases in this population. VSX1 is amemeber of the Vsx1 group of vertebrate paired-like homeodomian transcripition factors. It has been localized to the human chromosome 20p11-q11. *VSX1* is considered important in ocular development and is particularly involved in the developing cornea. Expression in human was demonstrated in embryonic craniofacial, adult cornea and adult retinal cDNA libraries [21]. *VSX1* mRNA has been found in the outer tier of the inner nuclear layer of the human retina and the cornea [22]. Thus far, mutations reported in this gene such as p.H244R, p.G160D, p.D144E, p.P247R, p.L159M, and p.R166W have been reported in various ethnic groups, but a definite pathogenic role has not been established [23]. *VSX1* mutations are also associated with posterior polymorphous dystrophy [24]. For these aforementioned reasons, the direct link of *VSX1* with keratoconus is controversial. This suggest that other loci, such as 13q32 [25], may be involved in the pathogenesis of keratoconus.

In our patient group, we noticed that females were more affected than males. In the literature, it is unclear whether significant differences between males and females exist. Some studies have not found differences in the prevalence between genders [7,26]; others have found a greater prevalence in females [27,28]; while other investigators have found a greater prevalence in males.

In the literature, most cases of keratoconus are sporadic, but a proportion (5%–10%) may be familial [6,7]. In our population, as judged by the family pedigree, 56.4% of cases were sporadic, 34.6% had autosomal recessive mode of inheritance, 5.4% were autosomal dominant, and 3.6% of cases were difficult to determine. So 40% (22 patients) of our keratoconus cohort were familial and this percentage is higher than that reported previously in the literature. This high rate of familial cases, could be attributed to the soaring scale of consanguinity in this society which reaches up to 60% in some areas of the Kingdom [29].

In the literature, 90% of pedigrees with familial keratoconus display an autosomal dominant inheritance with reduced penetrance [8,30]. Other modes of inheritance have been described, including autosomal recessive mode in families with children of consanguineous parents [31,32]. In our population, the familial cases (19 out of 22) had an autosomal recessive mode of inheritance. This is ideal for linkage analysis to identify the causative gene(s) in our population by focusing on families with multiple affected individuals, preferably in two or more generations.
